# The Effect of Participation Motivations on Interpersonal Relationships and Learning Achievement of Female College Students in Sports Club: Moderating Role of Club Involvement

**DOI:** 10.3390/ijerph17186514

**Published:** 2020-09-07

**Authors:** Chia-Ming Chang, Yu-Hui Chou, Huey-Hong Hsieh, Cheng-Kai Huange

**Affiliations:** 1 Department of Physical Education, Health & Recreation, National Chiayi University, Chiayi 62103, Taiwan; gr5166@yahoo.com.tw (C.-M.C.); a_ken5888@yahoo.com.tw (C.-K.H.); 2 Department of Recreation and Leisure Industry Management, National Taiwan Sport University, Taoyuan 333, Taiwan; alex.yh.chou@ntsu.edu.tw; 3 Department of Leisure Management, Taiwan Shoufu University, Tainan 72153, Taiwan

**Keywords:** female college students, participation motivation, interpersonal relationship, learning effectiveness, club involvement

## Abstract

The aim of this study is to explore the moderating effect of club involvement on the relationships of female college students’ sport club participation motivations for interpersonal relationships and learning achievement. Using cluster sampling, a structured questionnaire was distributed to 450 female college students located in northern, central, and southern Taiwan with a valid return rate of 96.2%. Using partial least squares structural equation modeling (PLS-SEM) analysis, the study found that the female college students’ participation motivations both affected interpersonal relationships and learning achievement positively. In addition, the moderating effects of club involvement on interpersonal relationships and learning achievement were both significant. Club involvement enhanced the effects of the female college students’ sport club participation motivations for interpersonal relationships and learning achievement. According to the results and discussion, practical application and future research suggestions were provided.

## 1. Introduction

Aside from study, college students can participate in sports clubs during leisure time. Among sports activities, students can increase sports knowledge, improve skills, train their body, and interact with people. Sports clubs provide an environment for students to release stress, improve physical fitness, and associate with friends. Therefore, sports clubs play an important role by shaping students’ personality and developing group cohesion, which can help each individual in the adaption to social lives [1].

Motivation is a driving force for sports club participation. According to Ryan and Deci [2], motivations can be classified into two parts, namely, intrinsic motivation and extrinsic motivation. Intrinsic motivation involves engaging in a behavior because it is personally rewarding (achievement, interests, curiosity, etc.); essentially, performing an activity for its own sake rather than the desire for some external reward [3]. Extrinsic motivation occurs when we are motivated to perform a behavior or engage in an activity to earn a reward (money, social status elevation, friendship, etc.) or avoid punishment [4]. In sports clubs, motivation is an important factor driving one to participate and stay in the club.

According to Yeh [5], participation in clubs is important for college students to establish social networks, and most students in clubs can improve communication skills, share time together, and when they assimilate into clubs, they may lead, obey, compete with, and cooperate with club members. Those are very helpful for them to adapt to workplace life [6]. In addition, by involvement in clubs, college students have good opportunities to promote interpersonal relationships with others as they can join the same activities and share time together [7]. Some studies have explored the effect of participation in clubs and organizations on the students’ development of mature interpersonal relationships. They concluded that the students’ participation extended their capacity for more mature interpersonal relationships by increasing their tolerance of and acceptance for other people and raising their confidence [8,9]. Based on the findings, we will explore the relationships among sports club participation motivation, involvement, and interpersonal relationships.

In colleges, most sports club members comprise more men than women as sport is commonly considered the domain of men [10]. Studies suggest that participation in sports clubs can cultivate exercise habits, improve exercise skills, and be satisfying [11]. Club learning effectiveness, as defined by Chung [11], is used to evaluate the participants’ progress in clubs, which comprise three domains, namely, cognitive, affective, and psychomotor domains. The higher the effectiveness, the higher the satisfaction, which will increase the enjoyment of participants in clubs. Therefore, we will also examine whether sports club participation motivations have influence on the learning effectiveness of female college students.

According to Havitz and Dimanche [12], involvement is defined as an unobservable state of motivation, arousal, or interest toward a recreational activity or associated product. It is evoked by a particular stimulus or situation and has driving properties. In other words, involvement refers to how we think about our leisure and recreation, and it affects our behavior as well. Based on the definition, once a person’s interest of participation in a sports club has been aroused, he/she will be more involved in the activity and then his/her learning effectiveness and interpersonal relationship will increase. Therefore, we will also examine the moderating effects of involvement between motivation and interpersonal relationships and between motivation and learning effectiveness among college sports club female student members.

## 2. Literature Review and Hypothesis Development

In this section, we will review the literature related to this study, which include: Relationship between participation motivation and interpersonal relationships, relationship between participation motivation and learning effectiveness, and the effects of club involvement among participation motivation to interpersonal relationships and learning effectiveness. Among the review, our research framework will be derived and established based on previous studies.

### 2.1. The Relationship between Participation Motivation and Interpersonal Relationship

In college, clubs can recruit people from different departments, and students can engage in organizing clubs’ activities and share time together [13]. Through clubs, they get to know more people from different fields and different backgrounds; hence, clubs are very attractive to students. Most students want to join at least one or two clubs to enjoy college life, and joining clubs can release some stress out of study [14]. Though everyone’s participation motivations may vary, they all learn interpersonal interaction and communication skills, which help them to build up good interpersonal relationships [15]. In addition, through engagement in clubs, members get to know each other, which can create social networks, enhance college and club identity, and increase cohesion [16]. Previous studies also found the positive relationships between participation motivations of sports clubs and interpersonal relationships [17,18]. Drawing from previous findings, participation motivation in sports clubs is found to be one of the key factors influencing interpersonal relationships. Thus, this study proposes the following hypothesis:

**H1.**  Participation motivation in sports clubs will positively affect female college students’ interpersonal relationships significantly.

### 2.2. The Relationship between Participation Motivation and Learning Effectiveness

Motivation is a driving force that can affect one’s behavior. One with strong participation motivation in a sports club will make efforts toward learning during the exercise process to satisfy one’s desire of learning more skills and knowledge [11]. Accordingly, the learning effectiveness of the higher motivators will be better than that of lower motivators [17]. Yang, Wan, and Wu [7] studied the relationship between college students’ club participation motivation and learning effectiveness. They concluded that motivation is positively related to learning effectiveness. Thus, this study proposes the following hypothesis:

**H2.**  Participation motivation in sports clubs will positively affect university female students’ learning effectiveness significantly.

### 2.3. Relationship of Club Involvement Among Participation Motivation, Interpersonal Relationship, and Learning Effectiveness

Involvement is an important factor influencing students’ interpersonal relationship development and learning effectiveness in clubs. Antil [19] proposed that club involvement is stimulated from a person feeling important and the degree of favoritism perceived by oneself to the club. Hu and Mou [20] studied the social network of teachers’ badminton club; they concluded that through regular involvement in tournaments, participants’ skills and interpersonal relationships improved significantly. Similarly, Wang and Chiu [1] studied the development value and function of sports clubs in colleges; they concluded that students with a higher level of involvement in clubs had better learning effectiveness. Chang, Shih, Kao, and Huang [21] studied the sports club and exercise participation behavior of college students, and they found that the female students’ participation frequency is positively related to learning effectiveness. As for athletes, Chao, Wu, Lin, and Wang [22] found that the players’ involvement can enhance the effect of participation motivation on satisfaction, and event support. Thus, this study proposes the following hypotheses:

**H3.**  The level of club involvement will significantly enhance the effect of participation motivation in a sports club on female college students’ interpersonal relationships.

**H4.**  The level of club involvement will significantly enhance the effect of participation motivation in a sports club on female college students’ learning effectiveness.

**Figure 1:**
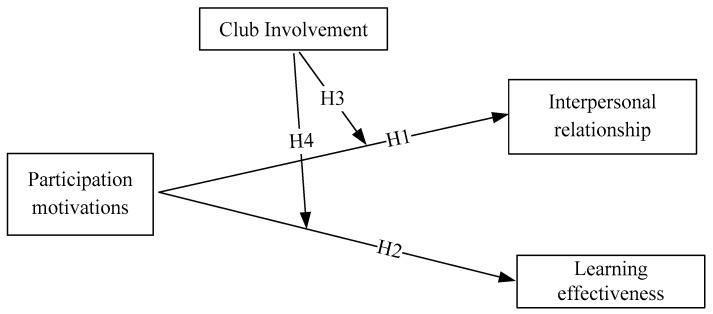
The proposed hypotheses.

Figure 1 presents the research hypothesis framework.

## 3. Methods

### 3.1. Participants

This study used a structured questionnaire for data collections. As female college students are the target subjects of this study, we had selected female sports club members as respondents from 15 universities located in the northern, central, and southern part of Taiwan. As for each university, we planned to collect information from three sports clubs’ female members, and 10 questionnaires were distributed to each club member, which, in total, summed up to 450 questionnaires. As for the collections, emails were sent directly to the dean of the selected university’s department of student affair to ask for approval for data collection, and a teacher/staff was asked to assist in the investigation as a tester. Exemption from IRB oversight was approved by the Ministry of Science and Technology, Taiwan, ROC. Before investigation, students all agreed to sign a consent form. Finally, we were able to collect 433 valid questionnaires with a return rate of 96.2%.

Table 1 presents descriptive statistics of the participants’ background variables. For the female participants’ basic information, regarding grade, junior subjects are the most (253 with percentage of 71.9%); for the type of sports club, the balls club is the most popular (191 with percentage of 58.43%); for the frequency of participation, 55.2% participate in activities once in a week, 26.33% participate 2–3 times per week, and 12.93% participate more than three times a week, which indicates that most of them are very active in clubs. For the position in the club, most of them are members solely (79.12%).

### 3.2. Survey Instrument

The survey instrument consists of three scales: Sports club participation motivation scale, interpersonal relationship scale, and learning effectiveness scale. The following sections will elucidate the formulation of the scales, and tests of the reliability and validity of the scales will be described as well.

#### 3.2.1. Sports Club Participation Motivation Scale

The sports club participation motivation scale was modified from the scales developed by Hsu, Hsieh, Lin, and Li [20], and Chang and Chen [23]. This is a measure of motives for participating in sports clubs comprising 11 items that consist of three constructs, namely, “health,” “knowledge,” and “social needs.” The items were measured on a 5-point Likert scale ranging from 1 (strongly disagree) to 5 (strongly agree). Participants were instructed that all items followed the stem “I participate in sport club…” Examples of the items are “because I want to increase physical fitness,” “to learn more sports skills,” and “because I enjoy spending time with others.”

The reliability and validity of the instrument were evaluated using partial least squares structural modeling (PLS-SEM). As for reliability, Fornell and Lacker [24] suggested the composite reliability (CR), and Cronbach’s alpha should be greater than 0.7. In our study, the CR of total scale was 0.89 and Cronbach’s alpha of total scale was 0.86, which indicated good reliability. As for convergent validity, Hair, Black, Babin, and Anderson [25] suggested that each item’s factor loading within a construct should be greater than 0.5, and the average variance extract (AVE) should be greater than 0.5. In our study, the factor loadings in the “health” construct ranged from 0.68 to 0.81; in the “knowledge” construct, they ranged from 0.64 to 0.74; and in the “social need” construct, they ranged from 0.56 to 0.63. The AVE was 0.44, a little bit lower than 0.5. In general, our test results indicated that the scale had good reliability and validity.

#### 3.2.2. Interpersonal Relationship Scale

The interpersonal relationship scale was modified from Wang, Chou, Peng, and Yeh [26]. This is a measure of interpersonal relationship for participating in a sports club comprising 8 items, which consists of two constructs: “Social skills” and “love for others.” The items were measured on a 5-point Likert scale ranging from 1 (strongly disagree) to 5 (strongly agree). Examples of the items are “During activities, I can always find friends to play with,” and “I often hang out with club members.” The CR of total scale was 0.83 and Cronbach’ alpha of total scale was 0.76, which indicated good reliability. As for factor loadings, “social skills” construct’s items ranged from 0.54 to 0.72 and “love for others” construct’s items ranged from 0.60 to 0.78, and the AVE was 0.41, a little bit lower than 0.5. In general, our test results indicated the scale had good reliability and validity.

#### 3.2.3. Learning Effectiveness Scale

The learning effectiveness scale was designated to measure students’ learning effectiveness in the participations of sports club. In this study, we adapted the effectiveness scale developed by Lee, Yang, Lin, and Chen [18], which was originally designed to measure junior high school students’ learning effectiveness in sports clubs. The scale consists of three constructs: “Cognition,” “affection,” and “psychomotor abilities,” which consists of 12 items and were measured on a 5-point Likert scale ranging from 1 (strongly disagree) to 5 (strongly agree). Examples of the items are “I can use some skills in daily life learning from the club,” “I learn how to cooperate with others from club,” and “I learned some motor skills from the club.” The CR of total scale was 0.91 and Cronbach’ alpha of total scale was 0.9, which indicated good reliability. As for factor loadings, “cognition” construct’s items ranged from 0.61 to 0.71; “affection” construct’s items ranged from 0.54 to 0.71; and “psychomotor abilities” construct’s items ranged from 0.70 to 0.76, and the AVE was 0.53. In general, our test results indicated the scale had good reliability and validity.

#### 3.2.4. Club Involvement Scale

In this study, the club involvement scale was modified from the college students’ club involvement scale developed by Hsieh [27] and rephrased to fit in the sports club involvement situation. There are two constructs in this scale: “Centrality” and “attraction,” which comprise eight items and were measured on a 5-point Likert scale ranging from 1 (strongly disagree) to 5 (strongly agree). Examples of the items are “I like to discuss sports club-related issues with club members,” and “Participating in the sports club is very enjoyable.” The CR of total scale was 0.903 and Cronbach’ alpha of total scale was 0.87, which indicated good reliability. As for factor loadings, “centrality” construct’s items ranged from 0.60 to 0.71 and “attraction” construct’s items ranged from 0.76 to 0.84, and the AVE was 0.46, a little bit lower than 0.5. In general, our test results indicated the scale had good reliability and validity.

### 3.3. Data Analysis

In this analysis study, PLS-SEM (partial least squares structural equation modeling) was used to test the hypotheses derived in the previous section. According to Chin [28], PLS-SEM benefits from (1) being distribution-free, (2) requiring only a small sample size, (3) having the ability to process multiple dependent and independent variables simultaneously, (4) handling collinearity, and (5) processing either formative or reflective indicators. Therefore, PLS–SEM was used to test the relationships in the study. There are many statistical software packages that enable users to perform PLS-SEM analysis. In this study, we used Warp PLS 6.0 developed by Kock [29] for data analysis.

## 4. Results

Reliability and convergent validity of the survey instrument was tested using PLS-SEM and confirmed in the previous section. The following section will present the discriminant validity descriptive statistics of all scales and test results of hypotheses using PLS-SEM, and, finally, we will compare the results with previous studies in the discussion section.

### 4.1. Discriminant Validity

Table 2 presents the test results of discriminant validity of the survey instrument. According to Fornell and Lacker [24], the discriminant validity is ensured when the inter-scale correlations are less than the scale’s square root of average variance extract (AVE). Our test results show that the inter-scale correlations are all less than its own square root of AVE, which indicated good discriminant validity.

### 4.2. Descriptive Statistics of Measurements

The descriptive statistics of all measurements is listed in Table 3. For participation motivations, the mean of the 11 items ranged from 3.69 to 4.27, which indicated that the female students had high participation motivations for sports clubs, and for skewness and kurtosis, all indicators showed a normal distribution. For interpersonal relationships, the mean of eight items ranged from 2.73 to 3.94, which indicated the female students’ interpersonal relationship was above average but not excellent (only one below 3). For skewness and kurtosis, all indicators showed a normal distribution. For club involvement, the mean of the eight items ranged from 3.15 to 4.09, indicating high involvement, and for skewness and kurtosis, all indicators showed a normal distribution. For club learning effectiveness, the mean of the 12 items ranged from 3.67 to 4.16, indicating high learning effectiveness, and for skewness and kurtosis, all indicators showed a normal distribution.

### 4.3. Hypotheses Test Results

**Figure 2:**
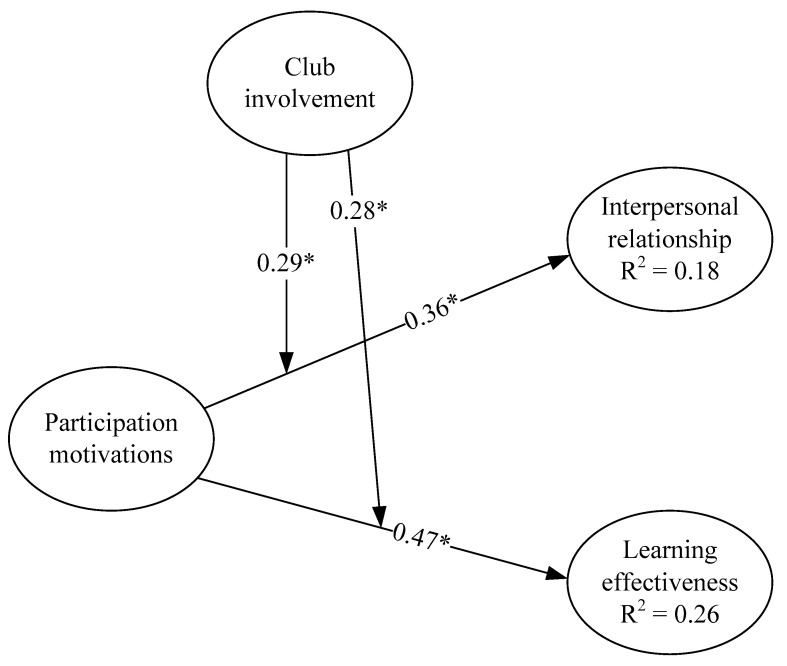
Structural equation modeling (SEM) results of the standardized model parameter estimation. Note: * *p* < 0.05.

Figure 2 presents the estimated standardized path coefficients (*β*) of the overall model. As we can see from the test results, all path coefficients are significant, indicating all hypotheses are supported. The following describes the test results of the hypotheses in detail:

**H1.**  Participation motivation in sports clubs will positively affect female college students’ interpersonal relationships significantly. The test results showed that the path coefficient β = 0.36, p < 0.05; therefore, the hypothesis is supported.

**H2.**  Participation motivation in sports clubs will positively affect university female students’ learning effectiveness significantly. The test results showed that the path coefficient β = 0.47, p < 0.05; therefore, the hypothesis is supported.

**H3.**  The level of club involvement will significantly enhance the effect of participation motivation in sports clubs on female college students’ interpersonal relationships. The test results showed that the path coefficient β = 0.29, p < 0.05; therefore, the hypothesis is supported.

**H4.**  The level of club involvement will significantly enhance the effect of participation motivation in sports clubs on female college students’ learning effectiveness. The test results showed that the path coefficient β = 0.28, p < 0.05; therefore, the hypothesis is supported.

### 4.4. Explanatory Power R^2^

R^2^ indicates the predictive power of predicting variables to the outcome variables. It refers to the predictive power of the research model. In the test results, participation motivations together with club involvement explained 18% of the variance in interpersonal relationships and 26% of the variance in learning effectiveness.

### 4.5. Discussion

#### 4.5.1. Participation Motivations and Interpersonal Relationship

This study found that female college students’ sports club participation motivations affect interpersonal relationships positively. This finding is consistent with previous findings [15,16]. Chen [30] pointed out that students considered participation in sports club activities as leisure after classes. Therefore, it is an autonomous choice for them to join activities so the atmosphere is full of joy and fun, which makes students want to do it over and over again. Lee, Yang, Lin, and Chen [18] also pointed out that students’ zeal for clubs come from the fun and elevated emotion during the time of activities. Their stress was released, and sharing together also satisfies their emotional needs, which motivates them to remain in clubs. Drawing from these findings, if sports clubs want to keep members’ zeal and participations, they need to have attractions and satisfy members’ emotional needs [31]. For example, they can invite popular coaches and celebrities, increase team cohesion, and arrange suitable activities comparable to the members’ ability, which can make their club attractive and satisfy members’ emotional needs.

#### 4.5.2. Participation Motivations and Learning Effectiveness

This study found that female college students’ sports club participation motivations affect learning effectiveness positively. This finding is also consistent with previous research [7,17], which pointed out that the participation motivations are the driving force of personal behaviors and will also affect learning effectiveness; and the spillover effect will further extend to academic achievement. For example, Kuo, Wang, and Lin [32] separated college students into club participants and non-participants and found that club participants’ academic achievements were better than non-participants. They concluded that club participants might release academic stress through the participations in sports club activities. Liu et al. [33] also pointed out that regularly engaging in physical activities can activate brain functions and, hence, promote learning effectiveness in every aspect. Stearns and Glennie [34] also pointed out that students in the level of middle engagement in clubs had better performance in academics. In summary, we suggest that students participating in at least one sports club can be beneficial to their learning effectiveness and achievement in every aspect.

#### 4.5.3. Club Involvement as an Enhancer

In our study, we found that club involvement can enhance the effects of sports club participation motivations on both the interpersonal relationships and learning effectiveness. Wang and Chiu [1] pointed out that the higher the involvement in clubs, the better their learning effectiveness. They found participants can gain confidence, make friends, and establish social networks through the involvement in clubs. Through the continuation in club participations, they will be willing to spend more time and make efforts in the club’s development. Especially, in sports clubs, it is easier to interact with people, make friends, and build social networks. Once students build good social networks, they can also get help and promote learning effectiveness [16].

## 5. Conclusions, Implications and Suggestions for Future Research

### 5.1. Conclusions

This study explored the effect of female college students’ sports club participation motivation on interpersonal relationships and learning effectiveness. Moreover, in this study, we examined whether club involvement had moderating effects among those factors. Our findings suggested that participation motivations had both positive effects on interpersonal relationships and learning effectiveness, and club involvement had enhancing effects among those relationships.

### 5.2. Implications

In Taiwan, people say that the three “must do” things for students in university are “academics, clubs, and romance.” Participation in a club is a good way to achieve these three together. We can find friends, lovers, have fun together, study together… There is so much fun to share and enjoy together. Meanwhile, involvements in clubs also train us to learn how to work together, how to manage an organization, and how to deal with people. There are so many benefits for students to be involved with clubs. As for sports clubs, there is a deep-rooted idea that sports is for men not for women (especially in oriental society). Therefore, we will provide three practical suggestions for club leaders and college administrators according to our findings as follows:

- Promotion of sports club participation motivation: According to our findings, as female college students’ sports club participation can affect their interpersonal relationships and learning effectiveness, the sports club itself can consider many aspects to attract female members, such as club orientations, promotion activities, and showcase of club achievements, especially, invitations of more female members to join the activities to deliver the fun image of sports clubs, which can certainly attract more female members.
- Offering funding to support sports clubs: As sports equipment is expensive, colleges’ administrators may consider offering extra funding for procurement and maintenance and set regulations for clubs to apply for funding, which can definitely attract more students to participate in sports clubs.
- Encouraging female students to participate in sports clubs: Our research findings suggest participations in sports clubs have positive effects on interpersonal relationships and can help students to build social networks, which will contribute to their career adaption and development. By encouraging those to participate in sports clubs in orientation or through instructors, it may motivate them to join sports clubs.
- Enhancing involvements in clubs: Our study found that club involvement can enhance the effects of sports club participants’ interpersonal relationships and achievement. Therefore, we suggest that sports clubs may provide sequential lessons for new participants to help them gain progress little by little and to get acquainted with other members. Once they feel more comfortable, they will love to spend more time with club members and play together, which will make them more confident and engage more in clubs.

### 5.3. Suggestions for Future Research

This study focused on all sports clubs but did not study which types of sports clubs attract more female students. Therefore, we suggest investigating which types of sports clubs may provide more satisfaction for female participants, which may also help the colleges’ administrators to develop/promote such types of sports clubs for female students.

## Figures and Tables

**Table 1:** Description of the demographic variables of the participants (N = 433).

Variables	Groups	N	%	Variables	Groups	N	%
Grade	Freshman	89	20.6	Frequency of participation	Once in a month	24	5.54
	Sophomore	89	20.6		Once in a week	239	55.20
	Junior	191	44.1		2–3 times per week	114	26.33
	Senior	64	14.8		More than 3 times per week	56	12.93
Type of sports club	Balls	253	58.43	Position in club	Leader or deputy leader	33	7.62
	Athletics	48	11.09		Staff	57	13.16
	Martial arts	49	11.32		Member	343	79.21
	Dancing	28	6.47				
	Leisure sports	17	3.93				
	Others	38	8.78				

**Table 2:** Test result of discriminant validity.

	(1)	(2)	(3)	(4)
(1) Participation motivations	0.66 ^a^			
(2) Interpersonal relationship	0.30	0.64 ^a^		
(3) Club involvement	0.35	0.54	0.73 ^a^	
(4) Club learning effectiveness	0.42	0.55	0.58	0.68 ^a^

Note: ^a^: Square root of AVE (average variance extracted).

**Table 3:** Descriptive statistics of measurement items.

Measurement Items	Mean	Standard Deviation	Skewness	Kurtosis
Participation Motivations (PM)	4.09			
PM1	4.26	0.64	−0.29	−0.70
PM2	4.27	0.72	−1.20	3.13
PM3	4.18	0.71	−0.44	−0.38
PM4	4.23	0.71	−0.51	−0.31
PM5	4.19	0.71	−1.07	2.92
PM6	3.98	0.75	−0.37	−0.14
PM7	4.07	0.75	−0.37	−0.43
PM8	4.18	0.74	−0.58	−0.07
PM9	3.87	0.98	−0.82	0.25
PM10	3.69	0.91	−0.46	−0.21
PM11	4.12	0.77	−0.58	−0.10
Interpersonal Relationship (IR)	3.62			
IR1	3.53	.89	−0.43	0.01
IR2	2.73	1.08	0.07	−0.70
IR3	3.74	0.84	−0.15	−0.60
IR4	3.82	0.82	−0.28	0.03
IR5	3.94	0.81	−0.76	0.95
IR6	3.63	0.90	−0.49	0.12
IR7	3.80	0.85	−0.69	0.88
IR8	3.72	0.83	−0.63	0.95
Club Involvement (CI)	3.69			
CI1	3.15	0.99	0.04	−0.74
CI2	3.50	0.95	−0.70	0.48
CI3	3.48	0.92	−0.39	−0.23
CI4	3.50	1.03	−0.44	−0.37
CI5	3.78	0.86	−0.47	0.11
CI6	4.09	0.79	−0.73	0.90
CI7	4.06	0.83	−0.79	00.80
CI8	3.96	0.79	−0.26	−0.61
Club Learning Effectiveness (CLE)	3.98			
CLA1	3.94	0.86	−0.67	0.38
CLA2	3.96	0.88	−0.74	0.42
CLA3	3.67	0.88	−0.71	0.89
CLA4	3.91	0.86	−0.89	1.27
CLA5	3.83	0.76	−0.21	−0.34
CLA6	4.07	0.79	−0.94	1.57
CLA7	3.99	0.75	−0.55	0.30
CLA8	4.13	0.77	−0.50	−0.38
CLA9	4.10	0.76	−0.73	1.07
CLA10	4.16	0.84	−0.81	0.43
CLA11	3.94	0.82	−0.71	0.76
CLA12	4.04	0.84	−0.75	0.65
